# Progress in Disease of Digestive Organs

**Published:** 1900-12-22

**Authors:** 


### Progress in Disease of Digestive Organs.

**Chylous Ascites.**—J. Halliday Croom[46] reports an instance due to cancerous obstruction at the junction of the mesenteric lacteals, while the thoracic duct was normal. A few cases have been due to rupture of the duct, but most often the condition is produced by obstruction. It is difficult to distinguish from ascites chyliformis or adiposus, where the milky fluid is said to be due to a degeneration of the serous endothelium, and contains sugar with large fat cells. The origin, however, of this fluid is disputed, and some writers say that even true chylous ascites may be lymphatic in origin.

**Dysentery.**—Much interest in this condition has been excited by the numerous outbreaks in the African army. Washbourn and O. Richards[47] received 250 cases in the Yeomanry Hospital: they classified them as (*a*) mild without pyrexia; (*b*) with pyrexia for a week and diarrhœa for a day or two before and after this; (*c*) attacks of tenesmus followed by pyrexia and diarrhœa for two or three weeks; (*d*) cases without tenesmus, blood, or mucus, but having pyrexia with diarrhœa or constipation and resembling typhoid, but distinguishable post mortem.

They advise treatment by ordinary ipecacuanha, magnesium sulphate, milk diet, and care in resuming a meat diet afterwards. In no cases did they find the amœba. Kenneth Macleod[48] urges the importance of washing dysenteric stools under a stream of water which carries off the blood and lighter portions of mucus, while the sloughs, clots, casts, and particles of undigested food are left behind for inspection. S. Flexner[49] agrees with Washbourn in accepting various sources and types of the disease, besides amœbæ, some specific bacilli, and other organisms are of importance. The bacillus seems a common cause, and resembles B. typhosus. A purpuric type of the disease is known, and shows little blood or mucus in the stools. Simonin[50] regards it as produced by various organisms normally parasitic, but which sometimes acquire virulence, and are then easily communicated to fresh patients. In war there are all the conditions under which they can attain this pathologic state. J. R. Wallace[51] recommends as an effective antiseptic treatment, an emulsion of castor oil, terebene, and opium morning and evening, 20 drops of the liquor hydrargyri every six hours, copious enemata containing boro-glyceride every twelve hours, and light solid food with fruit and vegetables.

**Food Stuff.**—Obesity is ably discussed by Van Noorden.[52] He points out that fat is largely derived from carbohydrates and proteids. Foreign fat is often burnt up and not stored. Obesity is lessened by (1) increase of exercise, and (2) by lessening the amount of food. Reduction of the amount of liquid may indirectly lessen fat, but the thyroid treatment seems to him undesirable. Ewald[53] confirms the view that in feeding by rectal enemata peptonised foods are not necessary, for the rectum actually absorbs egg albumen or that of milk in large proportions; so, too, starch and sugar are easily taken up. Fats present more difficulty; but even here he objects to pancreatised preparations, and after an elaborate review of the controversies on the subject he still recommends enemata made of flour, eggs, milk, glucose, and salt, though he would not wholly exclude such peptones as plasmon. Occasionally fat may be given by the slow injection into subcutaneous tissue of sterilised oil to the amount of 80 or 100 c.c.

Robt, under the direction of Ewald,[54] has carried out with great success a system of feeding exclusively by similar enemata. He gives a purgative enema in the morning followed by three nutrient ones during the day, amounting to 800 c.c. (25 oz.) The material is gently injected as high as possible, and the treatment is usually continued for six days under careful watching. It is chiefly applied in ulcer of the stomach, dilatation of the stomach, and intractable diarrhœa, so as to give absolute and entire rest to the stomach. Where hæmorrhage has been great it may be necessary to add large injections of saline solution to make up the fluid lost. The same holds good in impermeable cancer of the œsophagus, and indeed if symptoms of collapse and cardiac failure occur in any case saline injections should be substituted at once. When a week's rest for the stomach has been obtained, milk only is given by the mouth in teaspoonful doses, which are gradually increased. Afterwards purée of potatoes, pounded meat, and finally bread are added. In dilatation fluids, when given by the mouth, must be restricted, and solids should be supplied in small quantities at frequent meals. He claims some remarkable results, and the system has been elaborated with great care, and is minutely described in his paper.

[46] Lancet, June 23. [47] Brit. Med. Jour., Sept. 8. [48] New York Med. Jour., May 19. [49] Med. Rec., June 9. [50] Brit. Med. Jour., Sept. 18. [51] Dublin Jour. Med. Sci., August. [52] Brit. Med. Jour., Sept. 8. [53] Med. Rec., Aug. 18. [54] Dublin Jour. Med. Sci., August.

